# Identification of Risk Loci for Radiotherapy-Induced Tinnitus and Hearing Loss Through Integrated Genomic Analysis

**DOI:** 10.3390/ijms26094132

**Published:** 2025-04-26

**Authors:** Fan Ding, Zehao Pang, Xiujia Ji, Yuanfang Jiang, Qiulan Wang, Zhitong Bing

**Affiliations:** 1 Teaching and Experimental Training Center, Gansu University of Chinese Medicine, Lanzhou 730000, China; p11356557940421@163.com (Z.P.); jixiujia@gszy.edu.cn (X.J.); 18394422880@126.com (Y.J.); qiulwang@163.com (Q.W.); 2 Advanced Nuclear Physics Laboratory, Institute of Modern Physics, Chinese Academy of Sciences, Lanzhou 730000, China

**Keywords:** radiotherapy, tinnitus, hearing loss, genetic variants, PheWAS

## Abstract

Radiotherapy-induced hearing impairment significantly affects patients’ quality of life, yet its genetic basis remains poorly understood. This study seeks to identify genetic variants associated with radiotherapy-induced tinnitus and hearing loss and explore their functional implications. A genome-wide association study (GWAS) was conducted to identify single-nucleotide polymorphisms (SNPs) associated with radiotherapy-induced tinnitus and hearing loss. Protein–protein interaction networks and functional enrichment analyses were performed to explore underlying biological pathways. A phenome-wide association study (PheWAS) analysis across five databases examined associations between identified SNPs and various phenotypes. The GWAS identified 97 SNPs significantly associated with radiotherapy-induced tinnitus and 76 SNPs with hearing loss. Tinnitus-associated variants were enriched in pathways involving Wnt signaling and telomerase RNA regulation, while hearing-loss-associated variants were linked to calcium-dependent cell adhesion and neurotransmitter receptor regulation. The PheWAS analysis revealed significant associations between these hearing-impairment-related SNPs and metabolic phenotypes, particularly BMI and metabolic disorders. A chromosomal distribution analysis showed concentrated significant SNPs on chromosomes 1, 2, 5, and 10. This study identified distinct genetic architectures underlying radiotherapy-induced tinnitus and hearing loss, revealing different molecular pathways involved in their pathogenesis. The unexpected association with metabolic phenotypes suggests potential interactions between metabolic status and susceptibility to radiotherapy-induced hearing complications. These findings provide insights for developing genetic screening tools and targeted interventions to prevent or mitigate radiotherapy-related hearing damage.

## 1. Introduction

Pediatric brain tumors represent one of the most common childhood malignancies, with radiotherapy remaining a cornerstone in treatment [1,2]. While modern protocols have improved five-year survival rates to 75–85% [3], cranial radiation therapy (CRT) frequently leads to devastating complications, particularly radiation-induced hearing loss (RIHL) [4,5]. This complication significantly affects language acquisition and cognitive development and may seriously impact children’s growth, development, and learning abilities and even lead to social and emotional disorders [6]. While cochlear implants have significantly reduced tinnitus in those with hearing loss, the effect may lessen over time, pointing to the challenges of treating chronic hearing impairments [7]. The incidence of RIHL varies significantly with treatment parameters, affecting 22–35% of patients receiving high-dose CRT (54–56 Gy), particularly when combined with platinum-based chemotherapy [8]. Even at lower doses (30–36 Gy), approximately 15% of patients experience significant hearing deterioration [9,10]. Children under 7 years show particular vulnerability, with a higher increased risk of severe hearing loss compared to older cohorts [11].

The pathogenesis of pediatric RIHL involves complex molecular mechanisms, including oxidative-stress-induced hair cell death, microvascular damage, inflammatory responses, and impaired neural repair. Recent genome-wide association studies (GWASs) have identified several high-impact genetic variants, including ACYP2 [12,13] and WFS1 [14,15], suggesting strong genetic predisposition. However, the broader implications of these genetic findings remain unexplored. To address this gap, we present an integrated investigation combining GWAS and phenome-wide association study (PheWAS) approaches.

Our research extracted genetic loci information associated with hearing impairment in children after brain tumor radiotherapy, then used PheWAS analysis methods to examine the correlation between these genetic loci and phenotypic characteristics in five major phenotype databases. This analysis aims to elucidate molecular networks underlying RIHL with an emphasis on age-specific pathways, establish evidence-based risk stratification criteria, and identify protective interventions for high-risk patients. This research represents a crucial step toward personalized radiation oncology, potentially transforming treatment approaches for pediatric brain tumor patients through the integration of genetic and phenotypic risk factors.

## 2. Results

### 2.1. Identification of Genetic Variants and Functional Networks Associated with Radiotherapy-Induced Tinnitus Using GWAS Data

**Figure 1:**
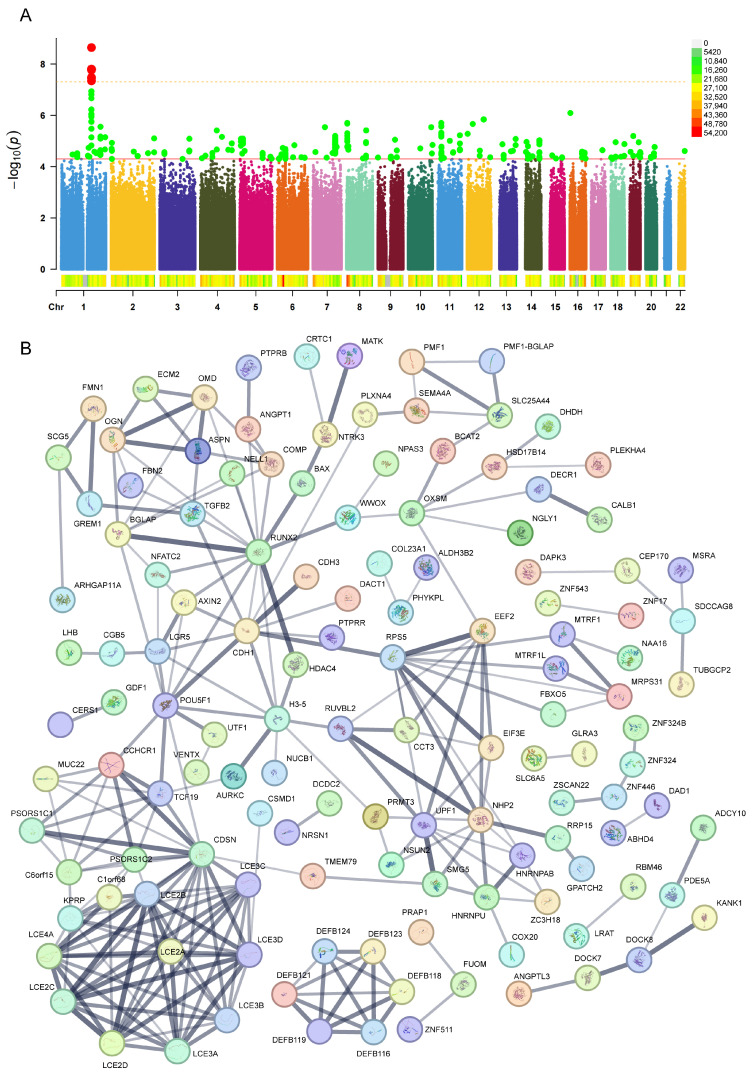
Distribution of genetic loci (**A**) significantly associated with tinnitus after cranial radiotherapy and the protein–protein interaction (PPI) network (**B**) of their proximal genes. (**A**) The distribution of genetic loci associated with tinnitus, with chromosomes 1–22 on the horizontal axis and −log_10_(P) on the vertical axis. Each dot represents a single-nucleotide polymorphism (SNP). Red dots indicate genetic loci significantly associated with tinnitus (P < 5 × 10^−8^, represented by the yellow dashed line at the top), while green dots represent loci with P < 5 × 10^−5^ (indicated by the second red solid line). The legend shows the distribution across chromosomal regions as indicated. (**B**) The PPI network of proximal genes, where each bubble represents a gene, and the black lines indicate the strength of interaction evidence between genes. Thicker lines represent stronger evidence of interaction.

Based on the established criteria (P < 5 × 10^−5^), 97 single-nucleotide polymorphisms (SNPs) significantly associated with radiotherapy-induced tinnitus were identified, with detailed information and neighboring genes influenced by each SNP provided in Table S1. Chromosomal distribution analysis (Figure 1A) revealed that these SNPs were dispersed across multiple chromosomes, with relatively concentrated significant SNPs on chromosomes 1, 4, 5, 6, 7, 8, 11, and 14. Notably, chromosome 1 exhibited multiple highly significant SNP loci (P < 5 × 10^8^). To understand the biological functions potentially influenced by these SNPs, a PPI network of the relevant neighboring genes was constructed using the STRING database. The results (Figure 1B) demonstrated significant interaction-enriched regions within these networks, primarily involving interactions among genes such as RUNX2, TGFB2, CDH1, RPS5, POU5F1, UPF1, NHP2, the DEFB family (including DEFB119, DEFB121, and DEFB124), CDSN, and the LCE family (including LCE2A, LCE2B, LCE2C, and LCE2D). These findings suggest that these genes may play important roles in the pathogenesis of radiotherapy-induced tinnitus.

**Figure 2:**
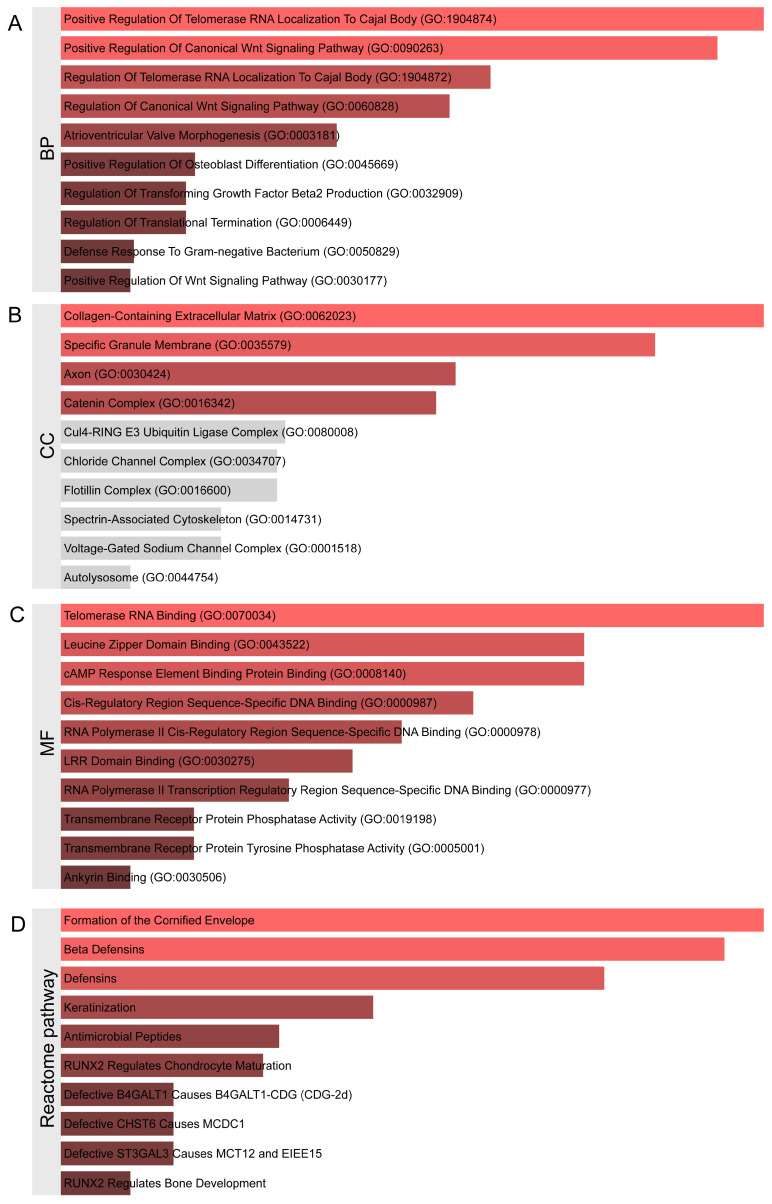
Functional enrichment analysis of proximal genes matched to genetic loci closely associated with post-radiotherapy tinnitus, where (**A**) represents biological processes, (**B**) represents molecular components, (**C**) represents molecular functions, and (**D**) represents Reactome pathway enrichment analysis. In the bar chart, red indicates an original *p*-value < 0.05, while gray indicates an original *p*-value > 0.05.

Subsequently, functional enrichment analysis was performed on these neighboring genes. Based on the Enrichr database, the analysis revealed extensive enrichment in biological processes (BPs) (Figure 2A, Table S2), including 81 statistically significant biological processes. The top 10 enriched terms were Positive Regulation of Telomerase RNA Localization to Cajal Body (GO:1904874), Positive Regulation of Canonical Wnt Signaling Pathway (GO:0090263), Regulation of Telomerase RNA Localization to Cajal Body (GO:1904872), Regulation of Canonical Wnt Signaling Pathway (GO:0060828), Atrioventricular Valve Morphogenesis (GO:0003181), Positive Regulation of Osteoblast Differentiation (GO:0045669), Regulation of Transforming Growth Factor Beta2 Production (GO:0032909), Regulation of Translational Termination (GO:0006449), Defense Response to Gram-negative Bacterium (GO:0050829), and Positive Regulation of Wnt Signaling Pathway (GO:0030177). The cellular component (CC) enrichment analysis (Figure 2B) identified statistically significant terms including Collagen-Containing Extracellular Matrix (GO:0062023), Specific Granule Membrane (GO:0035579), Axon (GO:0030424), and Catenin Complex (GO:0016342). The molecular function (MF) enrichment analysis (Figure 2C) revealed significant terms such as Telomerase RNA Binding (GO:0070034), Leucine Zipper Domain Binding (GO:0043522), cAMP Response Element Binding Protein Binding (GO:0008140), Cis-Regulatory Region Sequence-Specific DNA Binding (GO:0000987), RNA Polymerase II Cis-Regulatory Region Sequence-Specific DNA Binding (GO:0000978), LRR Domain Binding (GO:0030275), RNA Polymerase II Transcription Regulatory Region Sequence-Specific DNA Binding (GO:0000977), Transmembrane Receptor Protein Phosphatase Activity (GO:0019198), Transmembrane Receptor Protein Tyrosine Phosphatase Activity (GO:0005001), and Ankyrin Binding (GO:0030506).

The Reactome pathway enrichment analysis (Figure 2D, Table S3) primarily focused on Formation of the Cornified Envelope, Beta Defensins, Keratinization, Antimicrobial Peptides, RUNX2 Regulates Chondrocyte Maturation, Defective B4GALT1 Causes B4GALT1-CDG (CDG-2d), Defective CHST6 Causes MCDC1, Defective ST3GAL3 Causes MCT12 and EIEE15, Keratan Sulfate Degradation, Protein Methylation, and Signaling by NTRK3 (TRKC).

### 2.2. Identification of Genetic Variants and Functional Networks Associated with Radiotherapy-Induced Hearing Loss Using GWAS Data

**Figure 3:**
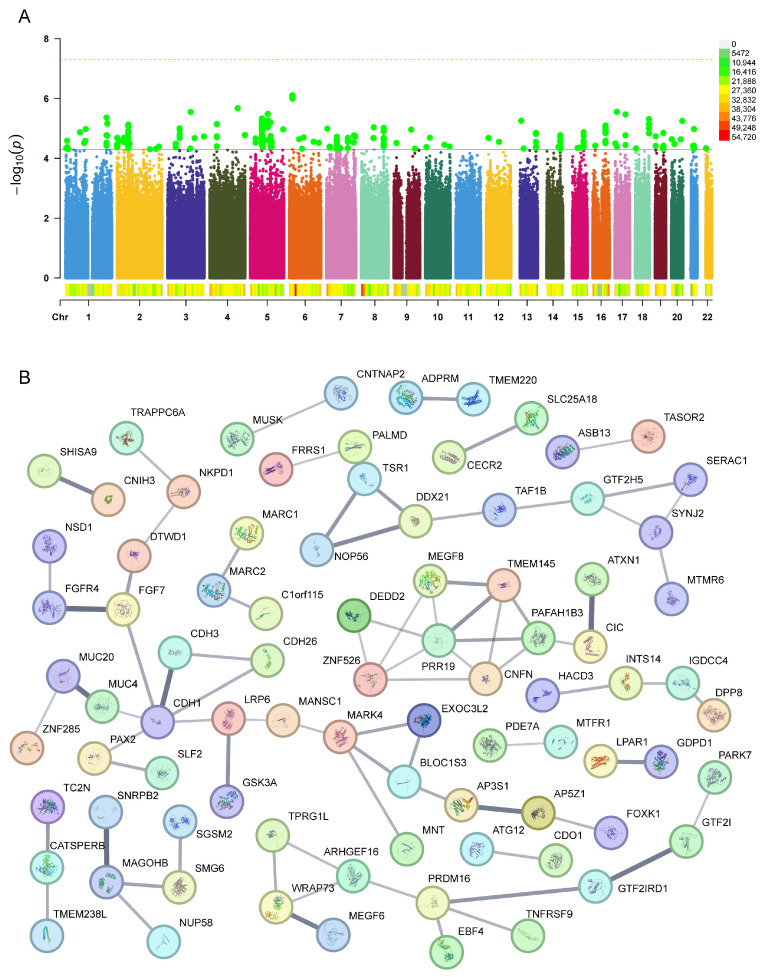
Distribution of genetic loci (**A**) significantly associated with hearing loss after cranial radiotherapy and the protein–protein interaction (PPI) network (**B**) of their proximal genes. (**A**) The distribution of genetic loci associated with hearing loss, with chromosomes 1–22 on the horizontal axis and -log_10_(P) on the vertical axis. Each dot represents a single-nucleotide polymorphism (SNP). Red dots indicate genetic loci significantly associated with hearing loss (P < 5 × 10^−8^, represented by the yellow dashed line at the top), while green dots represent loci with P < 5 × 10^−5^ (indicated by the second red solid line). The legend shows the distribution across chromosomal regions as indicated. (**B**) The PPI network of proximal genes, where each bubble represents a gene, and the black lines indicate the strength of interaction evidence between genes. Thicker lines represent stronger evidence of interaction.

Based on the established criteria (P < 5 × 10^−5^), 76 SNPs significantly associated with radiotherapy-induced hearing loss were identified, with detailed information and neighboring genes influenced by each SNP provided in Table S4. The chromosomal distribution analysis (Figure 3A) revealed that these SNPs were dispersed across multiple chromosomes, with relatively concentrated significant SNPs on chromosomes 1, 2, 5, and 7. To understand the biological functions potentially influenced by these SNPs, a PPI network of the relevant neighboring genes was constructed using the STRING database. The results (Figure 3B) demonstrated significant interaction-enriched regions within these networks, primarily involving interactions among genes such as CDH1, PRR19, and MAGOHB. Compared to tinnitus, the interactions between neighboring genes corresponding to hearing-loss-related SNPs did not display such prominent characteristics as observed in the tinnitus results. However, potential biological activities and regulatory processes of certain genes were still reflected. These findings support the potential important roles of these genes in the pathogenesis of radiotherapy-induced hearing loss.

**Figure 4:**
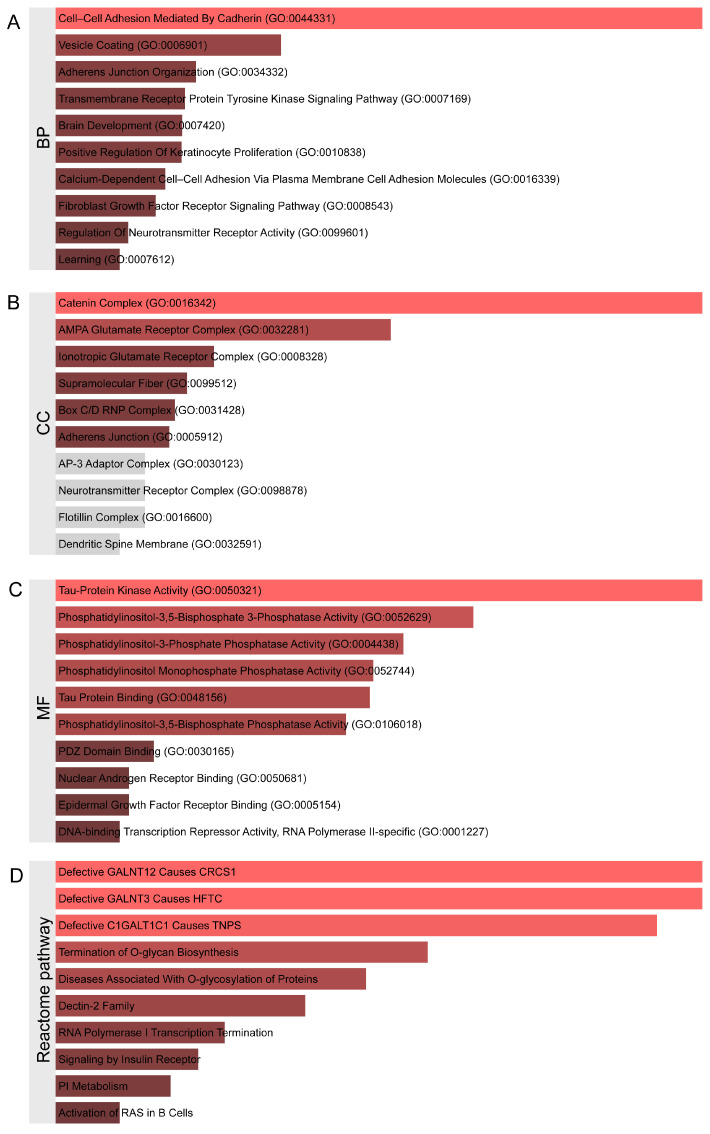
Functional enrichment analysis of proximal genes matched to genetic loci closely associated with post-radiotherapy hearing loss, where (**A**) represents biological processes, (**B**) represents molecular components, (**C**) represents molecular functions, and (**D**) represents Reactome pathway enrichment analysis. In the bar chart, red indicates an original *p*-value < 0.05, while gray indicates an original *p*-value > 0.05.

Subsequently, functional enrichment analysis was performed on these neighboring genes. The Enrichr analysis revealed extensive enrichment in biological processes (BPs) (Figure 4A, Table S5), including 81 statistically significant biological processes. The top 10 enriched terms were as follows: Cell–Cell Adhesion Mediated By Cadherin (GO:0044331), Vesicle Coating (GO:0006901), Adherens Junction Organization (GO:0034332), Transmembrane Receptor Protein Tyrosine Kinase Signaling Pathway (GO:0007169), Brain Development (GO:0007420), Positive Regulation Of Keratinocyte Proliferation (GO:0010838), Calcium-Dependent Cell–Cell Adhesion Via Plasma Membrane Cell Adhesion Molecules (GO:0016339), Fibroblast Growth Factor Receptor Signaling Pathway (GO:0008543), Regulation Of Neurotransmitter Receptor Activity (GO:0099601), and Learning (GO:0007612). The cellular component (CC) enrichment analysis (Figure 4B) identified statistically significant terms including Catenin Complex (GO:0016342), AMPA Glutamate Receptor Complex (GO:0032281), Ionotropic Glutamate Receptor Complex (GO:0008328), Supramolecular Fiber (GO:0099512), Box C/D RNP Complex (GO:0031428), and Adherens Junction (GO:0005912). The molecular function (MF) enrichment analysis (Figure 4C) revealed significant terms such as Tau-Protein Kinase Activity (GO:0050321), Phosphatidylinositol-3,5-Bisphosphate 3-Phosphatase Activity (GO:0052629), Phosphatidylinositol-3-Phosphate Phosphatase Activity (GO:0004438), Phosphatidylinositol Monophosphate Phosphatase Activity (GO:0052744), Tau Protein Binding (GO:0048156), Phosphatidylinositol-3,5-Bisphosphate Phosphatase Activity (GO:0106018), PDZ Domain Binding (GO:0030165), Nuclear Androgen Receptor Binding (GO:0050681), Epidermal Growth Factor Receptor Binding (GO:0005154), and DNA-binding Transcription Repressor Activity, RNA Polymerase II-specific (GO:0001227). The Reactome pathway enrichment analysis (Figure 4D) primarily focused on Defective GALNT12 Causes CRCS1, Defective GALNT3 Causes HFTC, Defective C1GALT1C1 Causes TNPS, Termination of O-glycan Biosynthesis, Diseases Associated With O-glycosylation of Proteins, Dectin-2 Family, RNA Polymerase I Transcription Termination, Signaling by Insulin Receptor, PI Metabolism, and Activation of RAS in B Cells. These results suggest that the aforementioned signaling pathways may play critical roles in the pathophysiological processes of severe hearing disorders following radiotherapy, providing an important theoretical basis for further elucidating the potential molecular mechanisms of radiotherapy-related severe hearing impairment and possible therapeutic targets.

### 2.3. PheWAS Analysis

#### 2.3.1. The Results of PheWAS Analysis for Phenotypes from Five Database

**Figure 5:**
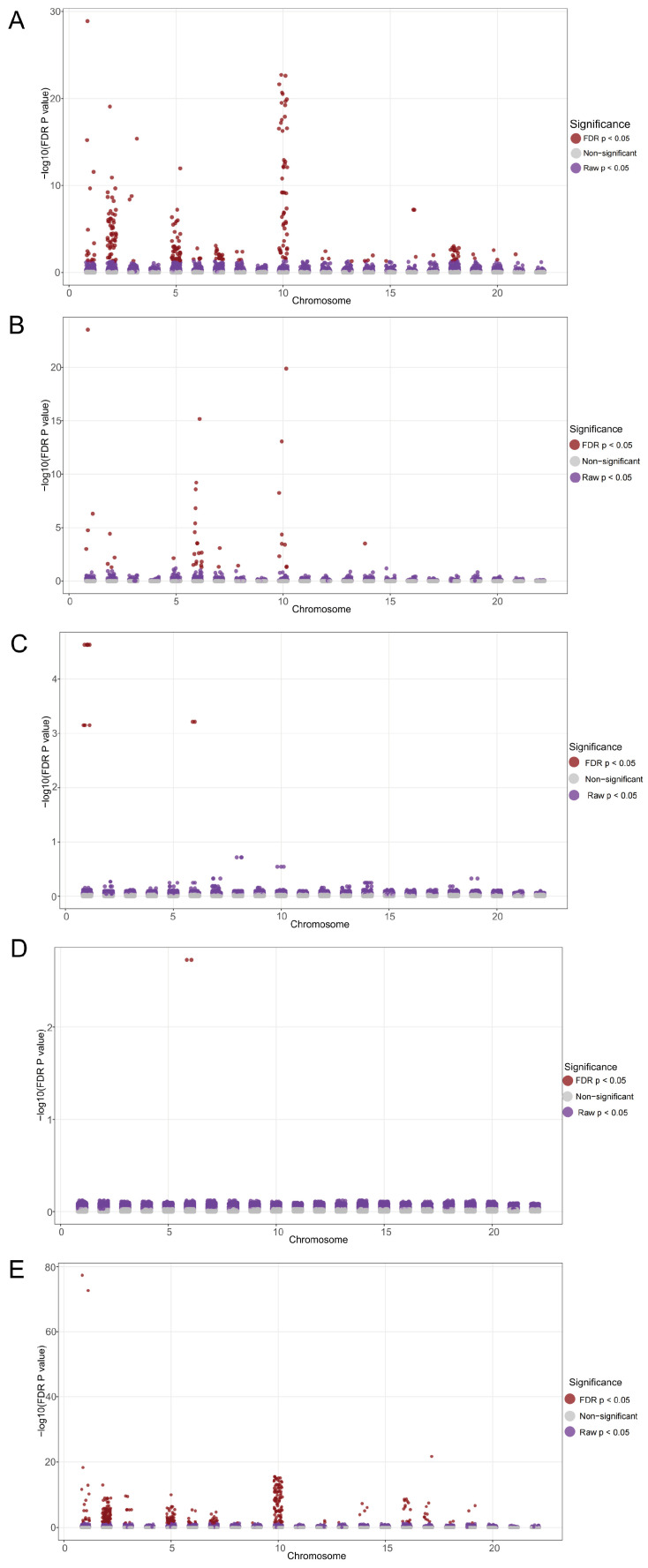
The PheWAS analysis identified the distribution of phenotypic correlations of genetic loci associated with radiotherapy-related hearing loss across five databases: (**A**) phenotypes from IEU UK Biobank; (**B**) FinnGen database (R11); (**C**) SAIGE UK Biobank; (**D**) TOPMed; (**E**) UKB BOLT. Each dot represents a single-nucleotide polymorphism (SNP). Brown dots indicate significant correlations between the current SNP and related phenotypes after FDR correction (FDR P < 0.05), while purple dots indicate correlations with raw *p*-values less than 0.05 between SNP loci and phenotypes.

We integrated a total of 173 SNPs associated with the two phenotypes and conducted a comprehensive PheWAS analysis across five phenotype databases from different populations. The results confirmed varying degrees of associations between these SNPs and different phenotypes (FDR P < 0.05, Figure 5). Specifically, significant associations were observed with phenotype data from the IEU UK Biobank (FDR P < 0.05, Figure 5A, detailed results are provided in Table S6), particularly for SNPs located on chromosomes 2, 5, and 10. Detailed results of these significant SNP–phenotype associations are provided in Table S6. The PheWAS analysis with phenotypes from FinnGen R11 also revealed a subset of SNPs demonstrating significant associations (FDR P < 0.05, Figure 5B, detailed results are provided in Table S7). Comparatively fewer associations were observed with phenotypes from SAIGE (FDR P < 0.05, Figure 5C, detailed results are provided in Table S8) and TOPMed (FDR P < 0.05, Figure 5D, detailed results are provided in Table S9). The analysis with the UKB BOLT data sources revealed numerous SNP–phenotype associations (FDR P < 0.05, Figure 5E, detailed results are provided in Table S10), similarly concentrated on chromosomes 2 and 10. These findings suggest that radiotherapy-induced hearing impairment may be more closely related to genetic loci on specific chromosomes, while also reflecting the potential impact of population heterogeneity and individual differences on the observed results.

#### 2.3.2. Category for Significant Phenotype Data from the IEU UK Biobank

**Figure 6:**
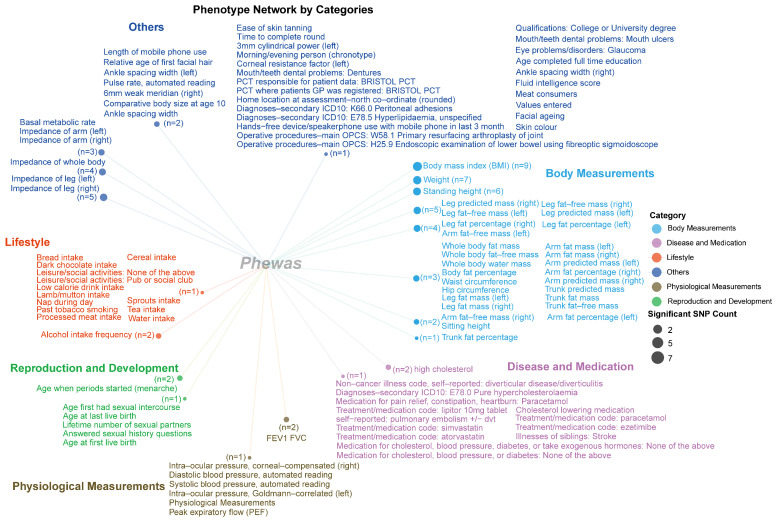
The PheWAS analysis identified the distribution of phenotypic correlations of genetic loci associated with radiotherapy-related hearing loss in the IEU UK Biobank database. Different-colored text and dots represent different phenotype classifications. The size of each dot indicates the number of significant single-nucleotide polymorphisms (SNPs) identified by the PheWAS analysis; larger dots represent a higher number of significantly correlated SNPs (for example, *n* = 9 indicates that there are 9 SNPs associated with these current phenotypes).

Based on the PheWAS analysis results, we categorized the significant phenotypes from the IEU UK Biobank database. The final results (Figure 6) showed that body-measurement-related phenotypes were associated with a larger number of SNPs. Specifically, Body Mass Index (BMI) was associated with nine SNPs, while weight was associated with seven SNPs. Additionally, body-fat-mass-related phenotypes, including fat mass measurements from different body regions, showed significant associations with 2–5 different SNPs. For other categories such as Disease and Medication, the associations were primarily observed in clinical medication use and comorbidity inverse correlations, with 1–2 SNPs per phenotype. Physiological measurements such as FEV1 and FVC also demonstrated significant associations with 1–2 SNPs. Reproduction and Development and Lifestyle-related phenotypes were associated with 1–2 significant SNPs each. Other phenotype categories, including impedance measurements of the leg or arm, showed associations with 3–5 significant SNPs, while medical-related phenotypes were associated with a single significant SNP.

#### 2.3.3. Category for Significant Phenotype Data from the FinnGen Database

**Figure 7:**
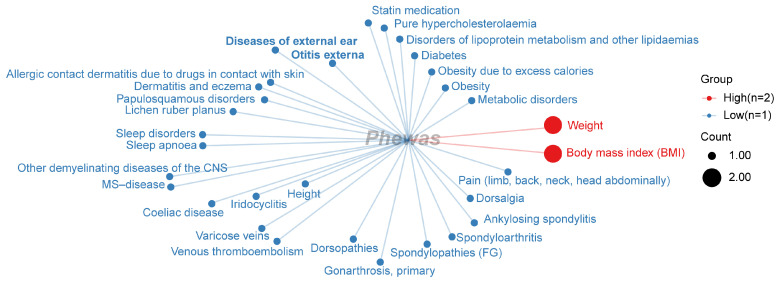
The PheWAS analysis identified the distribution of phenotypic correlations of genetic loci associated with radiotherapy-related hearing loss in the FinnGen database. Different-colored text and dots represent different phenotype classifications. The size of each dot indicates the number of significant single-nucleotide polymorphisms (SNPs) identified by the PheWAS analysis; red dots represent a higher number of significantly correlated SNPs (*n* = 2 indicates that there are 2 SNPs associated with these current phenotypes).

Based on the PheWAS analysis results, we categorized the significant phenotypes from the FinnGen database. As shown in Figure 7, weight and BMI were each associated with two significant SNPs, while other significant phenotypes were associated with only one significant SNP each. Among these phenotypes, we also identified ear-pathology-related traits, such as diseases of the external ear and otitis externa. However, in the PheWAS analysis of the FinnGen database, we did not observe significant phenotypes related to body fat mass, but instead found several metabolism-related phenotypic characteristics, including diabetes, obesity, disorders of lipoprotein metabolism, and other lipidemias, and pure hypercholesterolemia.

#### 2.3.4. Category for Significant Phenotype Data from the UKB BOLT Database

**Figure 8:**
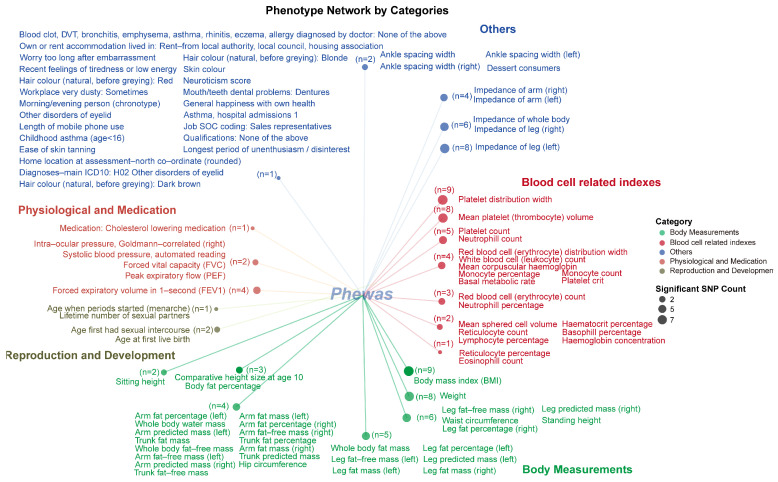
The PheWAS analysis identified the distribution of phenotypic correlations of genetic loci associated with radiotherapy-related hearing loss in the UK Biobank database. Different-colored text and dots represent different phenotype classifications. The size of each dot indicates the number of significant single-nucleotide polymorphisms (SNPs) identified by the PheWAS analysis; larger dots represent a higher number of significantly correlated SNPs (for example, *n* = 9 indicates that there are 9 SNPs associated with these current phenotypes).

Based on the PheWAS analysis results, we categorized the significant phenotypes from the UKB database. The final results are shown in Figure 8. Blood-cell-related phenotypes, such as platelet distribution width, mean platelet (thrombocyte) volume, platelet count, neutrophil count, monocyte percentage, and neutrophil percentage, demonstrated significant associations with 3–9 SNPs. Furthermore, in this data source, we also identified numerous significant associations with BMI (nine significantly associated SNPs), weight (eight significantly associated SNPs), and body-fat-mass-related phenotypes. Other phenotype categories, including reproduction and development-related traits, physiological and medication traits, and various other phenotypes, were each associated with 1–3 different SNPs.

## 3. Materials and Methods

### 3.1. Data Sources and Study Population

We obtained GWAS summary statistics for radiation-induced hearing loss and tinnitus from the GWAS Catalog database (accession numbers: GCST90027272 and GCST90027273) [16,17]. These data were originally generated from the Childhood Cancer Survivor Study (CCSS) and validated in the St. Jude Lifetime Cohort (SJLIFE). The tinnitus dataset represents a combined analysis of 2943 childhood cancer survivors of European ancestry (CCSS: *n* = 1991; SJLIFE: *n* = 952), while the hearing loss dataset includes summary statistics from 2529 individuals of European ancestry (CCSS: *n* = 2198; SJLIFE: *n* = 331). In the original studies [17], tinnitus and hearing loss outcomes were assessed through patient-reported questionnaires in the CCSS cohort, while the SJLIFE replication cohort used similar questionnaires for tinnitus and the Chang ototoxicity scale for hearing loss. All participants received cranial radiation therapy (CRT), and patients treated with cisplatin or carboplatin were excluded to isolate radiation-specific effects. The original study populations had a median age at primary cancer diagnosis of 8 years (range: 0–20 years) and median age at last observation of 43 years (range: 23–63 years). The most common primary diagnosis was acute lymphoblastic leukemia, representing approximately 28.5% of the study population. CRT doses varied widely, with survivors reporting tinnitus or hearing loss receiving a median maximum dose of 24 Gy.

### 3.2. Genetic Variant Selection and Processing

To identify genetic variants associated with radiation-induced hearing loss and tinnitus, we analyzed the two GWAS summary data obtained from the GWAS Catalog database. The data-processing pipeline included several quality control and filtering steps to ensure robust variant selection.

First, we standardized the column names and formats of the GWAS summary statistics. For variants reported as odds ratios (ORs), we converted these to beta coefficients using the natural logarithm transformation (β = log(OR)). Standard errors were calculated from the *p*-values and effect sizes using the formula SE = |log(OR)/qnorm(*p*-value/2)| [18], where qnorm represents the inverse of the standard normal cumulative distribution function.

To select variants significantly associated with our phenotypes of interest, we applied a liberal *p*-value threshold of 5 × 10^−5^ to capture potentially relevant signals while maintaining statistical stringency [19,20]. To address linkage disequilibrium (LD) and ensure independent signals, we performed LD clumping using PLINK (version 1.9). Variants were clumped using the following parameters: a clumping window of 1000 kb and an r^2^ threshold of 0.01 [21]. The 1000 Genomes Project Phase 3 European ancestry reference panel was used for estimating LD patterns, consistent with the ancestry of the discovery cohorts [22,23]. This process resulted in a set of independent genetic variants significantly associated with radiation-induced hearing loss and tinnitus that were used for subsequent analyses. The final variant set contained SNPs that were robustly associated with our phenotypes while minimizing correlation due to LD, making them suitable for downstream investigations of biological mechanisms and phenotypic associations.

### 3.3. Gene Mapping and Functional Enrichment Analysis

After identifying independent genetic variants associated with radiation-induced hearing loss and tinnitus, we performed functional characterization to elucidate the biological mechanisms underlying these associations. Using the R package “vautils” (version 0.1.0, https://github.com/oyhel/vautils, accessed on 10 February 2025), we mapped SNPs to their proximal genes based on genomic position. For each SNP, we identified the nearest genes within a 100 kb flanking region upstream and downstream using human genome build hg19 as the reference.

To understand the functional relationships between identified genes, we constructed a protein–protein interaction (PPI) network using the STRING database (version 12.0) [24]. Network parameters were set to include only interactions with a combined confidence score > 0.4 (medium strength), focusing on experimentally validated and database-curated interactions. This approach allowed us to visualize functional gene clusters and identify potential hub genes that may play central roles in radiation-induced ototoxicity.

Functional enrichment analyses were performed using the Enrichr database to identify biological processes, molecular functions, and pathways associated with our gene set [25]. We conducted a Gene Ontology (GO) enrichment analysis across three domains: biological processes, molecular functions, and cellular components [26]. Additionally, pathway enrichment analyses were conducted using Reactome databases to provide complementary insights into the relevant biological mechanisms [27]. For all enrichment analyses, we initially identified pathways with a nominal *p*-value < 0.05 [28]. While many of these associations did not remain significant after false discovery rate (FDR) correction for multiple testing, we reported both uncorrected and FDR-corrected *p*-values to provide a comprehensive view of potential biological signals. Pathways with an FDR q-value < 0.05 were considered statistically significant, while those with only nominal significance (uncorrected *p* < 0.05) were reported as suggestive findings that warrant further investigation. The combination of these approaches enabled the comprehensive characterization of the biological pathways and processes potentially involved in genetic susceptibility to radiation-induced hearing impairment, providing insights into the molecular mechanisms underlying this clinically important adverse effect.

### 3.4. Phenome-Wide Association Study (PheWAS) Analysis

To explore the broader phenotypic implications of genetic variants associated with radiation-induced hearing impairment, we conducted a PheWAS analysis. We used the set of independent SNPs (identified by their rs IDs) significantly associated with radiation-induced hearing loss and tinnitus as our input variants. The PheWAS analysis was performed across five major population-based biobanks and genomic databases with extensive phenotypic data: IEU UK Biobank (UKBB) (*n* = 463,005) [29], FinnGen R11 (*n* = 401,527) [30], UKBB SAIGE (*n* = 408,961) [31], TOPMed (*n* = 62,784) [32], and UKB BOLT (*n* = 487,409) [33]. This approach allowed us to examine associations between our hearing-loss-associated variants and thousands of phenotypes across multiple populations [34]. For each SNP–phenotype pair, we extracted comprehensive association statistics including effect size (beta), standard error (se), *p*-value, and trait information. Additional variant annotations were collected, including chromosome location, position, effect and non-effect alleles (ea, nea), variant consequences, and functional categories. Quality control metrics such as effect allele frequency (eaf) were also recorded. To account for multiple testing in our PheWAS analysis, we applied false discovery rate (FDR) correction [35]. Associations were categorized based on their statistical significance, with FDR-corrected *p*-values < 0.05 considered significant [36]. For each significant association, we recorded the phenotype code, detailed trait descriptions, and the source database file. This PheWAS analysis enabled us to identify potential pleiotropic effects of radiation-induced hearing loss variants across the human phenome, providing insights into shared biological mechanisms and potential comorbidities associated with genetic susceptibility to radiation-induced ototoxicity.

## 4. Discussion

This study employed GWAS methodology to explore the relationship between radiotherapy-related hearing impairment and genetic variants. The results revealed significant associations between radiotherapy-induced tinnitus and 97 SNPs, while hearing loss was significantly associated with 76 SNPs. The chromosomal distribution analysis showed tinnitus-associated SNPs primarily clustered on chromosomes 1, 4, 5, 6, 7, 8, 11, and 14, whereas hearing loss-associated SNPs exhibited a more dispersed pattern mainly on chromosomes 1, 2, 5, and 7. The protein–protein interaction network analysis identified prominent interaction characteristics among neighboring genes of tinnitus-associated loci (RUNX2, TGFB2, CDH1, and DEFB family) and hearing-loss-associated loci (CDH1, PRR19, and MAGOHB). The functional enrichment analysis indicated that neighboring genes of tinnitus-associated loci were primarily enriched in telomerase RNA localization regulation, Wnt signaling pathway, and cellular differentiation regulation. Conversely, neighboring genes of hearing-loss-associated loci were predominantly enriched in calcium-dependent cell adhesion, brain development, and neurotransmitter receptor activity regulation. The Reactome pathway enrichment revealed tinnitus-related keratinization and β-defensin pathways, while hearing loss was linked to glycosylation and glutamate receptor complexes, suggesting different pathophysiological mechanisms. The cross-database PheWAS analysis demonstrated significant associations between these hearing-impairment-related SNPs and metabolic phenotypes, particularly BMI and weight. The FinnGen database analysis identified additional metabolism-related characteristics, including diabetes, obesity, lipoprotein metabolism disorders, and hypercholesterolemia. The UKB BOLT analysis confirmed associations with BMI, weight, and body fat mass, while also revealing significant links to blood-cell-related phenotypes. This study revealed the potential genetic basis and molecular mechanisms of radiotherapy-related hearing impairment while elucidating connections between these impairments and metabolic phenotypes. These findings deepen our understanding of radiotherapy-related adverse reactions and provide a reference for developing individualized radiotherapy regimens in clinical practice.

From a clinical perspective, these findings hold substantial practical value for managing radiotherapy-induced hearing impairment. First, the identification of specific genetic variants associated with tinnitus and hearing loss following radiotherapy offers an opportunity for personalized risk assessment prior to treatment initiation. Clinicians could screen patients for these identified SNPs, particularly those on chromosomes 1, 2, 5, and 10, to stratify individuals at elevated risk for developing auditory complications and subsequently modify treatment protocols or implement preventive measures accordingly. Second, molecular pathways, including Wnt signaling and telomerase RNA regulation for tinnitus and calcium-dependent cell adhesion and glutamate receptor activity for hearing loss, represent promising targets for prophylactic interventions. Pharmacological agents that modulate these pathways could be administered concurrently with radiotherapy to mitigate ototoxicity while preserving therapeutic efficacy. Third, the PheWAS analysis revealing significant associations between hearing-impairment-related SNPs and metabolic phenotypes (including BMI, body fat mass, and metabolic disorders) suggests that baseline metabolic status may influence susceptibility to radiotherapy-induced auditory complications. This finding has immediate clinical implications for patient management, as optimizing metabolic parameters before initiating radiotherapy might reduce the incidence and severity of subsequent hearing disorders. Moreover, the differential molecular signatures between tinnitus and hearing loss provide a framework for developing targeted diagnostic tools to distinguish between these conditions at a molecular level, potentially enabling earlier and more precise intervention. Finally, the PPI networks identified in this study, particularly those involving RUNX2, TGFB2, and CDH1, may serve as the foundation for novel therapeutic approaches aimed at restoring auditory function in patients who have already experienced radiotherapy-induced hearing impairment. Collectively, these findings not only advance our understanding of the pathophysiological mechanisms underlying radiotherapy-induced auditory complications but also provide tangible avenues for improving clinical management through risk prediction, prevention, and targeted treatment strategies.

This study revealed risk genetic loci associated with radiotherapy-related hearing impairment through PheWAS and functional gene enrichment analysis methods, providing a new perspective for further understanding the pathophysiological mechanisms of radiotherapy-related hearing impairment from the angle of genetic variations. First, radiotherapy-induced tinnitus is closely related to biological processes such as the Wnt signaling pathway, telomerase RNA localization regulation, and osteoblast differentiation regulation, highlighting the important role of cellular damage repair responses in the occurrence of tinnitus after radiotherapy. The Wnt signaling pathway, as a key pathway regulating cell proliferation, differentiation, and tissue repair, may lead to the impaired repair of inner ear hair cells or spiral ganglion cells when abnormally activated, thereby triggering tinnitus symptoms [37,38,39]. Meanwhile, telomerase RNA localization disorders may affect the telomere maintenance and DNA damage repair capabilities of inner ear cells, making them more susceptible to apoptosis or functional impairment after radiation damage [40,41,42]. The significant interaction characteristics of genes such as RUNX2 [43,44] and TGFB2 [45,46] also suggest that abnormal bone and soft tissue remodeling may participate in the pathophysiological process of tinnitus, which is consistent with the degenerative changes in the bony labyrinth structure of the inner ear after radiotherapy.

Second, the neighboring genes of hearing-loss-associated SNPs are mainly enriched in processes such as calcium-dependent cell adhesion, brain development, and neurotransmitter receptor activity regulation, revealing the core role of synaptic transmission and neural signal transduction disorders in radiotherapy-related hearing loss [5,47,48,49]. Calcium ions play a crucial role in the mechanoelectrical transduction of inner ear hair cells and neuronal synaptic transmission, and abnormalities in their signaling pathways may directly lead to auditory signal transduction disorders [50,51]. Notably, the enrichment of glutamate receptor complex-related genes suggests that radiotherapy may induce excitotoxicity by affecting glutamatergic neurotransmission, ultimately leading to functional impairment or death of inner ear neurons [52,53,54]. Additionally, the enrichment of glycosylation-related pathways reveals the potential impact of abnormal post-translational modification of proteins in radiotherapy-induced hearing loss [55,56,57], which may be related to structural and activity changes of various functional proteins in the inner ear tissue, thereby affecting the normal function of the entire auditory system.

Third, this study, through the PheWAS analysis, for the first time revealed the extensive connection between radiotherapy-related hearing impairment and metabolic phenotypes, especially the significant associations with BMI, body fat mass, and metabolic diseases (such as diabetes and hyperlipidemia), suggesting that metabolic disorders may play an important role in the development of radiotherapy-related hearing impairment. Metabolic abnormalities may affect the severity of post-radiotherapy hearing impairment through multiple mechanisms, including (1) enhancing systemic inflammatory responses, amplifying local inflammatory damage to the inner ear caused by radiotherapy [58,59,60,61]; (2) aggravating oxidative stress, reducing the repair capacity of inner ear cells to radiation damage [62,63,64]; and (3) affecting microcirculation function, exacerbating inner ear vascular damage and ischemic conditions [65,66,67]. These findings not only explain the correlation between metabolic status and radiotherapy adverse reactions observed clinically but also provide new targets for the prevention and intervention of radiotherapy-related hearing impairment. In summary, this study revealed the multi-level pathophysiological mechanisms of radiotherapy-related hearing impairment through genetic approaches, including abnormal cellular repair responses, neural signal transduction disorders, and metabolic status influences, which interact with each other to collectively determine individual susceptibility to post-radiotherapy hearing impairment. These findings not only deepen our understanding of the molecular basis of radiotherapy-related hearing impairment but also give direction for developing prevention and treatment strategies, with the potential to achieve individualized hearing protection protocols by targeting these key pathways.

Despite the significant findings of this study, several limitations should be acknowledged. First, population heterogeneity represents a substantial constraint. The genetic variants identified in this study may exhibit different effects across diverse populations due to differences in genetic backgrounds, environmental exposures [68], and treatment protocols. The frequency and impact of these SNPs could vary significantly among different ethnic groups, potentially limiting the generalizability of our findings. Additionally, variations in radiotherapy delivery techniques, dosing schedules, and concurrent treatments among different clinical centers might introduce confounding factors that were not fully accounted for in our analysis.

Second, it is important to emphasize that the PheWAS analysis only established associations between genetic loci and phenotypes, which do not necessarily imply causality [35]. The significant correlations we observed between certain SNPs and hearing impairment, metabolic traits, or other phenotypes represent statistical relationships that require further validation through functional studies and prospective clinical trials. Without experimental validation, we cannot definitively determine whether these genetic variants directly contribute to radiotherapy-induced hearing impairment or merely serve as markers for other causal factors.

Third, this study was limited by the retrospective nature of the data collection and analysis. The assessment of hearing impairment outcomes was based on available clinical records, which may lack standardization in the evaluation methods and timing of assessments. While patients with platinum-based chemotherapy were excluded, several other important potential confounders were not fully accounted for in our analysis, including family history of hearing loss or tinnitus, variations in radiotherapy dose, specific areas of irradiation, age at treatment, and concurrent medications. These factors could significantly influence the risk and severity of hearing impairment, potentially affecting the strength and reliability of the observed genetic associations. The variability in defining and measuring tinnitus and hearing loss across different datasets could additionally introduce measurement bias and affect the reliability of phenotype–genotype associations. Furthermore, the lack of comprehensive longitudinal data on hearing function before, during, and after radiotherapy limited our ability to capture the dynamic progression of hearing impairment and its relationship with genetic factors over time.

Finally, while we identified numerous genetic variants and biological pathways potentially involved in radiotherapy-induced hearing impairment, the functional roles of many of these genetic loci remain unclear. The mechanistic connections between the identified SNPs and the proposed pathophysiological processes require further investigation through in vitro and in vivo experimental models. Our current analysis did not account for the potential impacts of immunotherapy and certain post-translational modification mechanisms on molecular pathways [69,70]. Additionally, our analysis did not fully address the potential gene–environment interactions and gene–gene interactions that could significantly modify the risk of hearing impairment following radiotherapy.

## 5. Conclusions

This study identified distinct genetic variants associated with radiotherapy-induced tinnitus and hearing loss through a GWAS analysis. The findings revealed that tinnitus was primarily associated with Wnt signaling and telomerase RNA regulation, while hearing loss was linked to calcium-dependent cell adhesion and neurotransmitter receptor regulation. The PheWAS analysis uncovered associations between these hearing-impairment-related SNPs and metabolic phenotypes, particularly BMI and metabolic disorders, suggesting a relationship between metabolic status and susceptibility to radiotherapy-induced hearing complications. Despite limitations including population heterogeneity and the associative rather than causal nature of the findings, this study provides insights for developing genetic screening tools to identify high-risk individuals before treatment, optimizing radiotherapy protocols, and designing interventions to prevent hearing damage.

## Data Availability

The datasets generated and/or analyzed during the current study are available in the IEU open GWAS project (https://gwas.mrcieu.ac.uk/, accessed on 10 February 2025), GWAS Catalog (https://www.ebi.ac.uk/gwas/, accessed on 10 February 2025), and FinnGen database (https://www.finngen.fi/en, accessed on 10 February 2025).

## Supplementary Materials

The following supporting information can be downloaded at: https://www.mdpi.com/article/10.3390/ijms26094132/s1.
